# "The Hospital" Nursing Section

**Published:** 1906-08-11

**Authors:** 


### The Hospital.

### Nursing Section.

Contributions for "The Hospital," should be addressed to the Editor, "The Hospital" Nursing Section, 28 & 29 Southampton Street, Strand, London, W.C.

No. 1,038.—Vol. XL. SATURDAY, AUGUST 11, 1906.

### Notes on News from the Nursing World.

### THE ROYAL RED CROSS FOR AN INDIAN NURSE.

It is officially announced that the King has conferred the decoration of the Royal Red Cross on Miss R. A. Betty, senior lady superintendent of Queen Alexandra's Military Nursing Service for India in recognition of her special devotion and competency during her long period of service in the Dependency.

### PRESENTATION OF A TESTIMONIAL TO MISS MONK.

A significant presentation has quietly been made to Miss K. H. Monk. On Monday last week Lord Methuen, Chairman of the Committee of Management of King's College Hospital, and a small deputation waited upon Miss Monk at her residence in Brighton for the purpose of presenting her with a testimonial on her retirement from the post of sister-matron after twenty-three years' devoted service to the hospital. The testimonial consisted of a purse of money, subscribed by upwards of five hundred of her personal friends, past and present members of the Committee of Management, and of the medical and the nursing staff of the institution. A book containing an address and a list of the names of the subscribers was also handed to Miss Monk.

### ORDERLIES AND ARMY SISTERS.

We publish on another page a letter which is extremely significant because it comes from an orderly belonging to the Royal Army Medical Corps. In an enclosure which the writer sends us he asks that blemishes of composition may be overlooked, because he does not pretend to be well educated, but expresses an earnest desire to correct the impression that the orderlies generally either entertain jealousy of the sisters or have any wish to disparage them. We make use of his communication the more gladly because it is so obviously dictated by sincerity, and because of the author's modesty. His testimony to the ability, the courtesy, and the patience of the female nurses will go far to remove the unpleasant impression created by a former correspondent, and to encourage the hope that, in spite of the ill-feeling of the few, sisters and orderlies may hereafter labour together with advantage for the benefit of sick and suffering soldiers

### THE ABSENCE OF NURSES ON MAIL STEAMERS.

Attention has appropriately been called in the holiday season by a former surgeon in the employ of the Royal Mail Steam Packet Company to the unsatisfactory medical and nursing arrangements on passenger vessels. With regard to the latter, he affirms that, under the "nurse-stewardess" and "hospital attendant" system, the third-class female passenger is entirely unprovided for. He thinks that the fact that she depends absolutely for nursing upon the surgeon and the male hospital attendant is "neither civilised nor decent." So do we, but this condemnation merely bears out the arguments which we have frequently used against the pretence of employing nurses on sea-going vessels. The "nurse-stewardess," even though she happen to be a trained nurse, will never occupy the position which it is essential she should possess if the nursing arrangements on passenger ships are to be in accordance with the requirements of civilisation and decency. We cannot understand the reluctance of the great steamship companies to follow the excellent example of the Booth Company. If they continue to persist in refusing to make proper arrangements for the nursing of passengers who, whatever class they travel, ought to be cared for in the event of illness by trained persons, it may become necessary for the Board of Trade, in the interests of humanity, to resort to compulsion.

### THE KINGSTON INFIRMARY STAFF.

It is very desirable that Poor-law Guardians should work harmoniously with the representatives of the Local Government Board, and we regret that one of the Kingston Guardians should have considered it advisable to say that, though they are subject to the control of the Local Government Board, they are not going to take their orders from an inspector. From all that we gather, the inspector in question, who recently visited Kingston Infirmary, fully recognised that the Guardians are simply anxious to have an adequate supply of nurses in order that the sick under their care, especially the children, may receive sufficient attention, and have no wish to augment the burden of the ratepayers unless it is absolutely necessary. We cannot conceive it probable that the Local Government Board will throw any obstacles in the way of increasing a staff when it can be proved that the increase is called for by the circumstances.

### AN UNFOUNDED CHARGE OF NEGLECT.

An extraordinary incident has occurred in the North of England. At a meeting of the Hayfield and New Mills Guardians last week one of the Infirmary nurses attended in reference to an allegation that a male patient in that institution had been neglected. Thereupon a Guardian, while denying that he had made a charge against the nurse, urged that the patient should have been in constant charge of a male attendant. He proceeded to describe the condition of the patient, provoking from the nurse the comment that he only showed his ignorance of nursing matters, and had not correctly stated the case. This view, the Chairman said, was endorsed by the medical officer, and in the result the Guardian who had complained of neglect withdrew his complaint, the Board passing a vote of confidence in the nurse. The Guardian, however, protested that there might always be "a wardsman to attend the sick." We do not think that his retrogressive theory will find much support.

### POISONED BY TINNED MEAT.

The announcement that twenty nurses at the Bellevue Hospital, New York, are suffering from ptomaine poisoning in consequence of having partaken of tinned tongue and ham suggests that there has been great carelessness in the housekeeping. The incident is the more inexcusable because of recent disclosures, and it can only be presumed that there are authorities at Bellevue Hospital who have not treated the Chicago scandals very seriously. It would be difficult to remove tinned meat entirely from hospital diet-lists, but before giving it to either nurses or patients the utmost care should be exercised in examination. We are glad to learn that the New York nurses are all recovering from the ill-effects of food which ought not to have been served out to them.

### NURSES AND MIDWIVES IN VICTORIA.

At a meeting of the Council of the Royal Victoria Trained Nurses Association held on June 19 three delegates, Misses Glover and Johnstone, and Dr. Meyer, were appointed to meet delegates from the medical societies to discuss the proposed Midwives' Registration Bill. The Council of the Nurses Association oppose the suggested legislation, unless the midwife is educationally and professionally required to be the equal of the present midwifery nurse—*i.e.* unless she has had three years' training in a general hospital and six months in a maternity school recognised by the Association. The Council suggest, for the better nursing of the poor, an externe department to the Women's Hospital, a midwifery branch connected with the present District Nursing Association, and, for those who can pay small fees, the visiting nurse.

### THE NEW INFIRMARY FOR CHILDREN AT LIVERPOOL.

We understand that Miss Ethel J. Atkins has been appointed the first lady superintendent of the new Infirmary for Children shortly to be opened in Liverpool. The more or less unusual duties of a lady superintendent in helping to organise and open a new institution of the sort will not be quite strange to Miss Atkins, as in 1897 she went as the first matron to the Cuddington Hospital in Surrey, and took up her duties there when only the bare walls of the building were standing. Since then Miss Atkins has had a wide and varied experience as nursing sister and acting superintendent of hospital ships during the South African War, and as matron of the Park Hospital, Hither Green, Shoreditch Infirmary, and the Royal Hospital, Portsmouth, the two latter being recognised training schools for nurses. Miss Atkins, who was trained at St. Bartholomew's Hospital, and holds the certificate of the Central Midwives Board, commences her new duties early in September.

### QUEEN'S NURSES AT WOLVERHAMPTON.

An interesting feature of the proceedings at the annual meeting of the Wolverhampton Queen Victoria's Nursing Institution was that a letter was read from the sister of a former patient enclosing a cheque for £5, in which she said that the life of her brother would probably have been lost but for the care of Miss Loveys, the matron, and her staff. This is a convincing proof of the value attached to the institution by individuals; its general popularity is attested by the fact that last year enough money was raised to enable the Committee to wipe off the remaining £100 of the mortgage. The importance of the district nursing branch is sufficiently indicated by a sentence from the report to the effect that 846 cases were treated and 17,227 visits paid, as against 597 cases and 11,387 visits during the previous twelve months.

### DISTRICT AND VILLAGE WORK IN CORNWALL.

The Cornish County Nursing Association is doing excellent work. There are now at work under its auspices nine Queen's nurses, five independently trained, 27 village nurses, and an emergency nurse who goes to any district where help is needed. Last year 3,531 patients were attended, including 440 maternity cases, and 136 operations. The District Association work entailed an expenditure of £2,857, and the County Association expended an additional £669, while its income amounted to £620, the deficit being met from funds in hand, exclusive of the £750 invested in stock. We are sorry, however, to learn that owing to lack of funds the idea of a joint supply of emergency nurses for the three Western counties has fallen through.

### POOR-LAW NURSING IN THE 'SIXTIES.

An excellent illustration of the progress which has been made in Poor-law nursing during the last forty years has just been afforded in Lancashire. Some old documents belonging to the Chorlton Union having been overhauled, reports were discovered which showed that in the 'sixties the Guardians were advised by the Poor-law authorities that a single highly-trained nurse was sufficient to have control of between eighty and ninety patients, so long as she was assisted by under-nurses selected from the inmates, in the proportion of one to every fifteen patients. At the same time they were informed "that the use of crinolines by nurses on duty should be strictly prohibited, it being a great hindrance to them in doing their work, and very inconvenient while passing between the beds." In the present day there is a paid nurse to every eight sick paupers, and the strength of the trained staff at Chorlton has thus been advanced about sevenfold.

### A HOME FOR EMERGENCY NURSES.

The Hon. Mrs. Stanley, wife of the late member for Bridgwater division of Somersetshire, was recently the recipient of an illuminated book and a cheque for £225 in recognition of her many acts of kindness to the town of Bridgwater and district. She has intimated that the money will be devoted to the inauguration of a home for emergency nurses in connection with the Somerset County Nursing Association. It will be called the Bridgwater Home, and will be started next spring. The gift is not only generous, but shows how thoroughly the donor understands the conditions on which district nursing can be most efficiently carried on.

### WOLVERHAMPTON EYE INFIRMARY.

Last week an interesting function took place at the Wolverhampton Eye Infirmary, when Miss Florence Newton, who was matron of the institution for fourteen years, was presented with a number of gifts in recognition of her services and the affection with which she was regarded by the staff. The Ladies' Committee gave her a gold watch; the Secretary, a gold chain; the nursing staff, ebony and silver brushes and glass; the domestic servants, silver salt-cellars; the gardener, a silver breakfast cruet; and the dispenser, a hand-painted satin table centre. Miss Newton, who is about to be married to the Rev. A. H. Lanfear, returned thanks for the gifts and for the compliments paid to her by the Chairman. Her successor, Miss Connell, is already at work, and carrying on her duties to the full satisfaction of the Management Committee.

### AN INCREASING DEBT AT OXFORD.

The District Nurses' Home in Wellington Square, Oxford—the Sarah Acland Memorial—is so well known and so much appreciated that it is surprising to learn that it does not meet with the financial support which it deserves. During last year the nurses paid 28,360 visits, were present at 43 operations, attended 161 confinements, and afforded relief in the case of 6,000 minor injuries. The cost of these services just exceeded £1,000, and the debt already existing was increased to £71. We notice that there are 200 subscribers, who contributed £265 18s. This is really a weak spot. In a prosperous city like Oxford the number of subscribers should be much larger. The Chairman of the committee states that it is necessary to look at every sixpence spent, but an augmentation of the income seems to be the most pressing need. Possibly, the Oxford people are under the impression that the Charity Trustees, who last year voted £190 to the fund, will come to its rescue. But the Charity Trustees already do all that is within their power, and it can hardly be expected that they will indefinitely continue such liberal support. The fact that at the present time the nurses attached to the Home are daily attending nearly a dozen patients suffering from cancer speaks volumes for the urgency of the work.

### POUND DAY AT TOTNES.

The first Pound Day in connection with Totnes Cottage Hospital coincided with the twenty-first anniversary of the opening of the institution, which has treated over 700 in-patients, and it was a gratifying success. One very welcome contribution was over £15 in cash, and, in addition, there were 53 lbs. of tea, 16 lbs. of sugar, 7 lbs. of tapioca, 14½ lbs. of biscuits, 10½ lbs. of cocoa, 42 lbs. of jam, 8 lbs. of coffee, 5 lbs. of arrowroot, 7 lbs. of cornflour, 6 lbs. of sweets, 6 lbs. of lard, 13 lbs. of dressings, 6 lbs. of fruit, 4 lbs. of sago, 5 lbs. of patent foods, 6 lbs. of butter, 6 lbs. of currants, 2 lbs. of vegetable wool, 2 lbs. of cake, 8 lbs. of rice, 4 lbs. of soap, 3 lbs. of vegetables, 18 lbs. of sundries, 12 bath towels, and 32 eggs.

### MIDWIFERY TRAINING IN NORFOLK.

At a recent meeting of the Norfolk Education Committee a report from a Sub-Committee, in favour of spending up to £250 during the next year on the training of midwives, was adopted. One of the speakers in support of the motion said that it had always been a difficult matter to provide adequate nursing in country parishes, and that the difficulty had been intensified by the operation of the Midwives Act. However that may be, the decision of the Norfolk Education Committee to spend a reasonable sum of money in twelve months upon the training of midwives can be unreservedly commended. There are other authorities which would do well to pursue a similar course.

### NURSING AMONG THE HOP PICKERS

In his annual appeal for monetary support, gifts of old linen for bandages, and illustrated papers in aid of the charitable, medical, and social work carried on by the Church of England Mission to Hop Pickers, the Rev. F. G. Oliphant, of Teston Rectory, Maidstone, states that last year the mission had 37 missionaries, 18 trained nurses, and 30 lady workers engaged in 28 parishes, paid and unpaid. This year there is every reason to believe the number, at any rate, of trained nurses, will be greater.

### MEMORIAL HOME AT WALSALL.

The nurses of the Walsall Victoria Nursing Institute have taken formal possession of the new Leckie Memorial Home, the gift of Mr. John Leckie, of Torquay, a retired Walsall manufacturer, in memory of his wife. A tablet in the hall of the building bears an inscription recording the fact as follows: "She personified in her gentle life goodness and kindness, and had the genius of friendship. Her sympathy for industrious families in sickness is sought to be perpetuated in this gift by her husband of forty-five years, in the hope that the temporary hopeless sick for ages to come may be tenderly cared for by the skilled nurses of this Christ-like institution." The total cost of the home was between £2,000 and £3,000. On the ground floor there is accommodation for special cases to be dealt with, a matron's room, a nurses' sitting-room, and dining-room; while upstairs there are eight bedrooms. Provision has thus been made for the much larger staff which, it is anticipated, will be required in the near future. With its adequate nurses' home, and its financial position placed on a sound basis by the bazaar which last autumn realised over £2,000 net, Walsall must be regarded as highly favoured.

### The Nursing Outlook.

"From magnanimity, all fears above;
From nobler recompense, above applause,
Which owes to man's short outlook all its charm."

### TRAINING FOR CHILDREN'S NURSES.

### III. The Liverpool Ladies Sanitary Association.

The training of children's nurses has been taken up by the Liverpool Ladies Sanitary Association in recent years, and the results so far attained are full of promise. The association drew up a scheme of training, and commenced with six students in January 1900. Since that time the work has gone on progressively, and at the end of five years the promoters have good cause for satisfaction. They have succeeded in opening a fresh means of honourable employment for educated women, and have brought safety and comfort into the nurseries of many homes. The special difficulty was to obtain for the students practical experience in nursery management and the general care of infants. It has not been found possible to have babies in residence at the training school, and heretofore the training of children's nurses in Liverpool has been secured by a three-months' course in the children's ward of a Poor-law infirmary, which constitutes the last three months of the training. The first three months are devoted to lectures on hygiene and the general management of children, including some simple lessons on housewifery; to lessons in the theory and practice of kindergarten for nursery use; to ambulance lectures by the St. John's Ambulance Association; to practical training in plain needlework, knitting, and millinery, with special reference to making children's clothes; and to the preparation of infant and invalid foods, and the washing of children's clothes. These classes are held partly at the Technical College of Domestic Science, and partly at the offices of the Association, Liverpool.

The gentlewomen students at the Liverpool Training School for Children's Nurses are enabled to obtain a six months' training, and to cover all expenses of board and training, for the sum of £30. At the end of six months a first certificate is granted to those students whose work has been satisfactory. On the completion of two years' service, if the reports from their employers are satisfactory, the nurses receive the final certificate and the badge of the Association, in the form of a silver brooch, and are then considered to be fully qualified. So soon as the first certificate is granted to a nurse she is entitled, and expected, to wear the uniform of the Association, which consists of a plain, dark-green bonnet and cloak. Recognising that true education begins in the nursery, the managers of the Liverpool School have so organised their system of training as to impress upon the nurses that the mental, moral, and physical characteristics of the child must always receive their due share of her consideration, and that the nurture of the young includes both the care of the mind and of the body. The Association rightly holds that a good children's nurse should possess a genuine love of children, tact, patience, good temper, and sound health. Candidates are therefore carefully tested during the first month, and, should they be found unsuitable for the work, the fees are returned to the student, and she is informed that she has no vocation for the work.

We have stated already that in the majority of middle-class homes it is not possible for the parents to pay large salaries to the nursery staff. The remuneration of the Liverpool nurse student is, however, fairly satisfactory, for at the end of six months she can obtain a post with a salary ranging from £20 to £25 a year; and at the end of her two years, when she becomes a fully qualified, experienced children's nurse she may reasonably expect to receive from £30 to £40 a year. If she is willing to go abroad her salary may amount to £60 a year or more. So much is said nowadays about the overcrowding of all departments of women's work, that it is satisfactory to be able to state, on the authority of the experience of the Liverpool Ladies Sanitary Association, that the demand for children's nurses trained under their system is undoubtedly in advance of the supply, and that the salaries of their certificated students are steadily improving.

Every intelligent parent must recognise the advantage of entrusting their children to the care of a nurse who brings to her task not only affection, but a trained, disciplined mind. Every children's nurse who is really interested in her work finds ample scope for all her love, energy, and originality in the efficient management of a nursery of young children in a middle-class home.

We hope that one result of the publication of our articles on this subject will be to direct the attention of young parents especially to the importance of securing a nurse for their children who has had an adequate training on the lines and for the reasons we have explained in the three consecutive articles on training for children's nurses. We have already started a special column for advertisements by trained nurses and other children's nurses and their employers. We hope the new department of this paper now devoted to the care and management of children will fulfil many useful purposes and be largely used by our readers and the public also. The more these subjects are discussed, the better will it be for children and parents alike. Ignorance is the curse of English children, and we shall do our part to remove it as far as practicable.

### Abdominal Surgery.

By Harold Burrows, M.B., F.R.C.S., Assistant Surgeon to the Seamen's Hospital, Greenwich, and to the Bolingbroke Hospital, Wandsworth Common.

### AFFECTIONS OF THE LIVER AND GALL-BLADDER (*continued from p.* 245).

### Infection of Gall-bladder and Bile-ducts.

It has been pointed out in a previous article that the bile-ducts and gall-bladder sometimes become infected by micro-organisms derived from the bowel into which the bile-duct opens. The effects of such bacterial invasions will vary according to the relative virulence of the micro-organisms and the duration of their pathological activities.

In the slightest cases no recognisable effect is produced. In others there is a temporary swelling of the mucous membrane of the duct, which is sufficient to prevent the outflow of bile, and so causes jaundice (catarrhal jaundice). In severer instances, suppuration may occur in the gall-bladder (suppurative cholecystitis), and the infection may lead to gangrene or perforation of the affected organ and acute peritonitis.

Little consideration need be given here to these results because the milder cases are not regarded as surgical affections, and because the acute suppurative and gangrenous cases, in their general symptoms and treatment, resemble other acute intra-abdominal infections, such as acute appendicitis, which have been dealt with already. These acute infections in nearly all cases require prompt operative treatment.

### Gall-stones.

But, from a surgical point of view, the most important complication of biliary infection is the formation of gall-stones.

Gall-stones are soft solid concretions which form in the gall-bladder. They give rise to repeated attacks of inflammation of the gall-bladder, and they may also cause trouble by escaping from the gall-bladder into the bile-duct, and so causing severe pain (gall-stone colic) and perhaps obstructive jaundice. For any cause which prevents the flow of bile from the liver to the intestine will produce jaundice. If a stone becomes arrested in the cystic duct (see illustration on p. 244) it will not cause jaundice, because the bile can still flow from the liver, where it is formed, along the hepatic and common bile-duct into the duodenum. But if the stone is lodged in the common bile-duct it is likely to prevent the escape of bile into the bowel, and jaundice will result. At the same time the stools will be pale, while the urine will contain bile pigment. The white of the eye is the place in which slight degrees of jaundice can be most easily perceived. Gall-stone colic is usually very painful; but, unlike that of peritonitis, the pain comes and goes, or, if it is constant, yet its intensity varies periodically. The pain is in the right upper part of the abdomen and may also be felt in the region of the right shoulder-blade.

In a case supposed to be gall-stone colic, as in all other cases of severe abdominal pain, the patient should be kept in bed, all food withheld, and a doctor summoned without delay. When the diagnosis has been made, the treatment will consist of rest in bed, hot fomentations over the affected region, and perhaps morphia or other sedative if the pain is too severe or the patient very restless. A dose of calomel may be required, and other drugs that are sometimes used are belladonna, olive oil, ammonium chloride, and sodium salicylate. Meantime a careful watch is kept for any abatement of the jaundice and alteration of the other symptoms. Disappearance of bile pigment from the urine and return of the normal colour to the stools are useful indications which should never be overlooked.

A watch should be kept also for the passage of the gall-stone by the bowel. This is done by washing the fæces through a fine-meshed sieve, or repeatedly washing them in a large vessel. A good method is by having some stout, wide-meshed gauze stitched on to a circle of iron wire which is fitted round the pan of the water-closet. The washing may be done in this way without great inconvenience by means of the ordinary flushing apparatus.

There are two reasons which render it good practice to search for the stone in this way. In the first place it is important to know if a gall-stone has been present, and the appearance of a stone in the fæces may be the only available evidence which is absolutely conclusive. In the second place, it is usually possible to perceive by an examination of the stone whether it was the only one present in the gall-bladder, or whether it was accompanied by others. If other gall-stones are present, the one that has been passed will show flat areas or facets which are due to the pressure of adjacent stones. The patient will be liable to subsequent attacks of colic and to other dangers so long as there are any gall-stones present. And as the only sure way of removing gall-stones is by operation, it is clear that the character of a concretion passed by the bowel may settle a problem whose correct solution is of vital interest to the patient—namely, whether he ought or ought not to undergo an operation.

I have been anxious to emphasise this matter because it is always an encouragement for those who have unpleasant duties to perform to know that their work is not only necessary but is of great importance to the patient.

The following are the operations performed on the gall-bladder: (1) Removal of the gall-bladder (cholecystectomy); (2) the making of an opening into the gall-bladder through which any concretions are extracted and by means of which drainage of the biliary system is provided (cholecystotomy); (3) incising the bile-duct in order to extract a stone (choledochotomy); and (4) the establishment of an artificial opening between the gall-bladder and intestine, so that if the common bile-duct is obliterated bile can still flow from the liver to the intestine (cholecystenterostomy).

### Abscess of the Liver.

Abscess of the liver is most frequently secondary to infection in some other abdominal organ. As a complication of dysentery so-called tropical abscess of the liver is not rare. In dysentery the mucous membrane of the large intestine is apt to become ulcerated. By means of the ulcers microbes are enabled to enter the patient's blood. Having entered the blood they are carried by the portal vein to the liver, in the capillary vessels of which they become arrested, and so form the commencement of an abscess in the liver.

The symptoms are pain and tenderness in the region of the liver, temperature of sepsis, general malaise, and ill-health; and, of course, a history of recent dysentery in conjunction with these symptoms is suggestive of hepatic abscess.

The treatment is similar to that of abscesses in other regions of the body, and consists of opening and draining the abscess. If this is not done in time the pus may burst into the right lung or into the peritoneal cavity, with dire results.

### The Nurses' Clinic.

### NOSE AND THROAT CASES—APPLICATIONS AND DIET.

There are many applications, external and internal, for the treatment of nose and throat cases, with which the nurse should be familiar, and know how to apply.

The most generally used applications are heat and cold.

There are various ways of applying heat, namely, with hot-water bottles or bags, sponges squeezed out of boiling water, hot fomentations, and hot bran or salt bags, and poultices.

The most effective and lasting means of applying heat is by fomentations or poultices. If it is possible to keep a hot-water bag or bottle near the poultice it will keep the heat in much longer.

Cold is best applied to the head and neck by ice-bags or coils, and to the nose by ice-cloths very frequently changed.

For allaying pain or inflammation soothing lotions may be applied, such as lead and opium, by cloths soaked in the solution.

To cause counter-irritation, blisters, mustard leaf, or tincture of iodine would be used, applied in the usual way.

Splints are used for fracture of the nose, and the nurse's duty is to see that they do not slip out of position. Splints for the nose are used externally and internally. The external splint is kept in place by strapping and a bandage.

The internal splint, which is generally made of hard, hollow rubber tubes, is kept in place by resting on the free lower edge of the nostril.

In giving a pharyngeal douche for clearing the throat or preparing it for operation, the tongue should be depressed, the solution being allowed to flow freely in the mouth, then to the palate, and lastly to the throat. In this way the throat can be cleansed without the patient swallowing a drop of water.

In giving a nasal douche, make the patient thoroughly understand that he must make no attempt to swallow, or breathe through the nose while the solution is running in, and then there will be no danger of him choking.

The head must never be bent backwards. The stream of water must be gentle and not swift or strong, or the water will enter the eustachian tubes, and may do great harm. The mouth, through which all the breathing must be done, must be kept wide open all the time. The nose must be dried by blowing into the handkerchief after the excess of water has been allowed to run out.

The nose and throat may be cleaned by medicated spray. Into the atomiser bottle put the solution ordered. Introduce the end of the spray into the nostril, pressing the bulb till the stream is started; continue this from fifteen to thirty seconds, then remove the spray into the other nostril and spray in the same manner. For the throat a straight spray tube should be used, the tongue being depressed and the point of the tube turned downwards.

When possible the patient should cover his tongue with a piece of lint and pull it forward. The nozzle should then be introduced into the mouth to within a quarter of an inch of the back of the throat. When the bulb is being pressed the patient should be told to inhale.

To spray the larynx effectually needs much practice, and is not accomplished at once—and will probably be given by the surgeon himself. Internal applications to the nose will be made with a camel's-hair brush or a swab on holder.

Ointment is introduced into the nose on the end of a swab, which is pushed into the nose. While the nostrils are firmly pressed from the outside of the nose, the swab is withdrawn, leaving the ointment in the nose.

The throat is painted with a swab, the tongue being depressed meanwhile. The holder should be bent to get the swab well down the throat. Powder is blown in by the usual powder blower. The patient should exhale while the powder is being blown in.

Inhalations will be given in one of the special inhalers. The drug is put into the vessel, which is filled up with boiling water, and the nozzle put into the mouth to let the steam get well to the back of the throat. Gargles are not so much used these days as they were formerly, as it has been found that as a rule the lotion does not go far enough back in the throat to do any real good. They are useful for cleansing the mouth out.

After operations on the nose and throat care in the diet may save the patient much discomfort.

For the first twenty-four hours the patient should be kept on light liquid diet, such as milk, beef tea, chicken broth, strong soup, tea or coffee, which should be given cold or cool.

If any fever sets in the patient must be kept on liquid food till the temperature is normal, six to eight ounces being given every three hours, but less to a child.

In nose operations diet is not so important as in throat operations, except in cases of operation upon the antrum through the mouth.

The diet in this case will be better given through a tube, so arranged that no fluid enters through the mouth into the antrum or nasal cavity. When the patient is put on more substantial diet, the antrum drainage-tube should be plugged, or he should chew the food on the unoperated side of the mouth. Food entering the antrum would remain there and decompose. After tonsil and adenoid operations, patients do not require much food, but what they have should be liquid. If patients have much pain in swallowing it will hurt them less if before swallowing they put a piece of ice in their mouth, and to take a large mouthful is less painful than a small sip. Ice-cream, which is very nourishing, may be taken when nothing else can be swallowed.

**THE NURSES' CLINIC—*Continued.***

In intubation cases, children should lie on their backs, the head being lower than the chest, in which position they can swallow large quantities of liquid nourishment.

In cleft palate some surgeons order the patient to be well fed up before the operation, and starved for a day or two after, not even allowing water. If the patient is very weak, and cannot go without food, it should be administered by nutrient enemata. After three days the patient may take liquid nourishment in small quantities through the mouth and small doses of water. The incision must be carefully watched, and if no harm arises from the diet it may be gradually increased. Solid food should not be given till three or four days after the stitches have been removed.

After removal of the epiglottis there is considerable difficulty in keeping the food out of the larynx. This can be accomplished by allowing the tongue to sink well back into the mouth while swallowing, when it makes an artificial epiglottis, closing the opening of the larynx and forcing food into the œsophagus. This method should first be tried with plain water.

### Incidents in a Nurse's Life.

### A CHILDREN'S NURSE.

Now that an effort has been made to encourage hospital nurses to take up duties as children's nurses, perhaps those who are thinking of doing so may think my experience interesting.

I had spent several years in a "home for sick children," but on leaving I found that I was not strong enough to enter a general hospital for training. At this time I obtained, through my former matron, a post to take entire charge of a delicate boy of three. Beyond being nervous and excitable, there was very little wrong with him. He was bright and intelligent for his age, and had suffered more from over-care than from neglect. His parents, though sensible people in other ways, were so anxious that he should only do such things as would be likely to benefit him, and to eat nothing but wholesome food, that although so young, the child would ask if "such-and-such a thing were good for him" before he took it, and the first time he was given a sweet he did not know what to do with it!

We had two nice rooms for day and night nurseries. These I kept tidy myself, but the housemaid always did the grate and helped with the weekly turnout, doing windows, brasses, etc., and all scrubbing. I kept the child out of doors as much as possible, and, of course, had the windows open day and night when we were indoors.

With fresh air and food suited to his age the boy soon grew stronger, and became more child-like. But I found the daily walks very monotonous, and after the busy life to which I had been accustomed there did not seem sufficient work.

After about nine months a baby boy appeared on the scene. On his arrival my duties increased, and, incidentally, my salary also. I now had the entire charge of both children, and as the baby had to be brought up on the bottle, my previous experience with infants proved most useful.

"His Majesty," the baby, grew and flourished on a mixture of milk (sterilised), water (boiled), lime-water, sugar, and extract of malt and oil. I mixed the food night and morning, and then had only to heat the required quantity at feeding-time. He had two teaspoonfuls of sugar, and the same of malt and oil, in the 24 hours—the milk, etc., varying according to his age. At a year old the bottle was quite discarded in favour of bread-crumbs and gravy, semolina pudding, and so on. At a year and ten months, when I left, he was a strong, healthy child, of whom, I think, we were all proud.

During the whole time I acted as children's nurse I met with the most unfailing kindness and consideration from the parents.

We hear much of the ignorance of the poor with regard to their children, and also their gratitude, but I think that the rich are almost as ignorant and quite as grateful.

I am sure that any nurses not strong enough for hospital work would do well to turn their attention to this branch of nursing, always provided, of course, that they are fond of and thoroughly understand, children.

Naturally it is not all sunshine, but no one expects that anywhere, and some of my happiest days have been spent with the little ones. The chief drawback, in my opinion, to becoming a children's nurse is the monotony; but to make up for this, in my case there was far less work than I had been accustomed to do, and that my health very greatly improved.

Beyond going to church once on Sundays, there was very little time "off duty," as it were; but my young charges were always asleep in good time, and then I was glad to sit still with a book or some needlework. I did all the children's mending, and made most of their garments, with the help of a machine. My meals were brought up to the nursery, excepting tea in the summer, which we usually had in the garden.

### The Treatment of Burns.

BY A DISTRICT MATRON.

In a long experience one sees many modes of treating these all too common accidents, but the old-fashioned use of carron oil is probably still the most usual to begin with. But in one hospital to which I went after training we had a totally different method, and one which was much cleaner and quite as effective, if, indeed, not more so.

For a first dressing we soaked linen (the doctors in that hospital much preferred linen to lint) in a solution of carbonate of soda, about an ounce of carbonate of soda to a pint of water, and applied it to the surface of the skin, covering it over with wool in the ordinary way, but the bandages were arranged so as to take off easily, either "esmarch" or "many tail" being generally used.

The wool was from time to time removed and the linen damped from the outside with the soda solution, so as to keep it constantly wet, without removing it, and this treatment was carried on for two or three days. If any part of the dressing became soiled with the discharge that part was removed and a fresh piece of soaked linen applied. Afterwards carbolised or eucalyptus vaseline was generally applied. We never washed a burn or exposed it to the air for a second longer than was necessary.

In applying the ointment dressing we wiped the discharge away with a piece of lint soaked in oil as we rolled the soiled dressing back, and as the old dressing was rolled off the new dressing was unrolled to replace it, so that only a fraction of the wound was exposed at a time, and that time of the very shortest duration.

It was marvellous how well burns and scalds treated in this way got on, and the carbonate of soda which was supposed "to take the burn out" was certainly most soothing in its effect.

In the later treatment of burns we not infrequently varied the ointments, but washing or exposing the wounds were always avoided. There was one case of a woman burnt by falling asleep while reading in bed and upsetting the lamp, which I particularly remember because it was unusual in its later treatment in two ways: First, the burns were grafted with egg skin; secondly, it was healed with chalk ointment. The burn extended all round the patient's left side from the sternum to the backbone, and as far down as her waist. She went on very well at first, and then the healing became very slow; so the surgeon for the case determined to graft.

Accordingly one afternoon I was sent for from tea, and told he wanted me. When I entered the ward he literally danced down to meet me, holding something in his hand, and repeating "I heard it cackle myself, so I know it's fresh."

In his excitement he had forgotten to take off his hat, which was tilted backwards and a little on one side, and my first impression was that the man had gone quite mad. It appears that he had been out for a walk a little way out of town and had heard a hen cackle, whereupon he rushed in to the astonished housewife and told her her hen had laid an egg and that he must have that egg. So he purchased it on the spot and came straight to the hospital in triumph. "Shall I send for the house surgeon?" said I. "No, there's no need to disturb him. We can manage all right by ourselves," he replied; so I proceeded to take off the dressing and wipe away the discharge with a clean piece of lint while the surgeon cut a small piece of egg skin off and applied it. He put on five pieces altogether covering them with green protective. I then dressed the side with ointment as before.

There was a great deal of discharge, so that it was necessary to dress the burn twice a day. At the first dressing two of the grafts came off, at the second two more, and at the third the last one came off, but in spite of that the healing began and continued steadily and swiftly from each of the five points where the grafts had been.

"I hope you won't grow feathers," said the surgeon as he left the patient.

The other point of interest with regard to this case was the use of chalk ointment, which I then used for the first time. Afterwards I employed it a good deal, and found it a clean and pleasant ointment and very healing in its effect. The last case in which I used it was a vitriol burn on a child's leg, and I had a good deal of difficulty in procuring it, and then it was a different preparation from what we used in hospital, and though it did very well was softer and not so pleasant to use.

In the country I have seen carbonate of soda soaked in vinegar applied to scalds, but it fizzes in a most alarming manner, and I should think the acid of the vinegar would annul the alkali of the soda. Still the result seemed satisfactory.

### Nursing in Industrial Schools.

When I was attached to a private nursing institution in the Midlands, about one o'clock one cold drizzling morning I was roused from my slumbers, and informed that I was to go to the Boys' Industrial School, at the other end of the town, to nurse a case of typhoid fever.

Hastily dressing, and putting a few necessary articles into a handbag, I was soon on my way, with the institution porter for an escort, a cab not being procurable at that untimely hour.

As soon as I arrived at the Industrial School I was shown into the sick-room, situated in an isolated block away from the main building, and approached by an outside staircase. A more dismal-looking place it has seldom been my lot to enter!

My patient was a boy of twelve, an inmate of the school, who lay, flushed and restless, on a little narrow bedstead. He was delirious, muttering incoherently.

The doctor, I was informed, had left orders that the patient was to be tepid sponged if the temperature rose over 103½°. After delivering the doctor's message the woman who was in attendance took her departure, and I was left alone with my patient.

My heart sank as I gazed round my quarters. A small tin lamp gave forth a fluttering light, revealing a long bare room, furnished with a wooden table and a chair. Two empty bedsteads stood in a corner, as well as the one occupied by my patient. The fire had gone out. Subsequent investigation showed me a room leading out at one end, which contained a few pots and pans and a cold water tap. At the other end was a lavatory, which I found had no water supply.

I must confess that I felt horribly inclined to sit down and have a good cry, but recollecting that my old matron's last words to me on leaving my training school had been "Always make the best of everything, nurse," I plucked up my courage, and proceeded to take my patient's temperature. This I found to be 104°, so I realised that I had work to do, which was the best thing that could happen to me. I soon found material for making a fire, and in a short time had sponged my patient, given him a drink, and had the satisfaction of seeing the restlessness decrease.

I then hunted round on a voyage of discovery, and at last found a bottle of Calvert's carbolic, which I made into a solution, and with this flushed the lavatory, washed utensils, and sprinkled it round generally and generously.

My spirits rose as the morning dawned. At 6 a.m. the silence was suddenly broken by the sound of a bugle, and out into the yard scrambled some dozens of the schoolboys. Off came jackets and shirts, and ablutions were performed at a row of taps. Judging from the blueness of the faces, and the grunts and gasps which accompanied the splashing, the water was cold. Poor little chaps! My heart ached for them, but—as I learnt afterwards—bowls of hot bread-and-milk were awaiting them directly their toilet was completed, so that matters might have been worse. Later on my watch was relieved by the daughter of the head of the school, she eventually proving of great assistance in taking duty during my hours of rest and recreation.

My patient got on splendidly. There was no further spread of the disease; and, in spite of many drawbacks and many lonely nights, I spent a not unprofitable month at Industrial School Number One.

. . . . . . .

Some months had elapsed. I was in the Home waiting my turn to go out, when the telephone bell rang, and in a few minutes the matron appeared.

### NURSING IN INDUSTRIAL SCHOOLS—*continued.*

"A case for you, nurse, at M—— Industrial School," mentioning a town some miles off.

"What, another?" I said, rather surprised. "Evidently matron, my mission in life must be in the direction of industrial schools."

I was soon on my way, and in due course arrived at my destination. I found the building to be a large one, accommodating 200 boys from the slums of several of the large towns of the Midlands, with a staff for teaching them various arts and crafts, in addition to the three R's.

This time I had four patients: three of them down with influenza; the other an unhealthy-looking boy of ten, with abscesses in various parts of his body, and a necrosed ankle.

The sick-room was a distinct improvement on the one at the last Industrial School at which I had nursed, being new and more up to date. There was a small kitchen attached, fitted with a diminutive range, a plentiful supply of hot and cold water, and all necessary crockery and utensils. I took duty by day, and we had a local "Sairy" for the night-watches. The "flu." boys were very ill indeed for some days, with high temperatures, and at one time we feared pneumonia, but happily that they escaped.

The abscess boy was a terror. He did not even catch the "flu." to keep him quiet. His bad leg was fixed on a long back-splint with footpiece, and whenever I turned my back, out of bed came the "imp," as I dubbed him, and I would hear the bump, bump of his splint across the floor. His delight was generally to torment either one or other of the unfortunate occupants of the other beds. My remonstrances were always met with the rejoinder, "I was just tidying him a bit, nurse," or "I only wanted to cheer him up," or some such remark, whilst an impish smile would overspread his features. When I did manage to keep him in bed he occupied the time by making balls of his splint-padding, to be shied when my back was turned, or by picking holes in his bedclothes. The doctor threatened all manner of punishment, and contrition was profound for a moment; then the old smile would appear again, as expansive as ever.

The epidemic of influenza lasted some time, and I had a good number of the boys through my hands, some only getting slight attacks, and consequently up to all sorts of mischief, but others needing much care and attention.

The doctor decidedly had a sobering influence on the obstreperous boys. He was a splendid type—tall, austere, brusque, but with a kindly nature and a fund of quiet humour under a rough exterior. Many a poor patient in the poverty-stricken quarter of M—— has been the richer for timely help in the shape of money or food after a visit, unknown to any but the recipients of the kindness. A look from the doctor worked wonders on the minds of those boys, and was an effectual check.

After a time, and when my charges were convalescent, I used to have a sort of out-patient department. All who had sores, cut fingers, toothache, or any other ache or pain were sent up to the small room we called the "haven" at a certain hour each day, and I attended to their wants. Malingerers, of course, there were in plenty. A day in the sick-room was a luxury, and many were the dodges devised in order to gain the coveted "sick-room leave."

During my ten weeks' sojourn at the School I had repeated opportunities of finding that many a brave, honest, and upright nature was to be found amongst the boys whom those in happier circumstances would dub "the children of the slums."

### The Ethics of Fresh Air.

### Fresh Air Never Causes Chill.

"One man's meat is another man's poison." This apparently was the view of a nurse, who, though advocating fresh air for herself, considered the same commodity detrimental to the well-being of her patient. As she explained to a patient under her care, "You are in a low state of health, and the treacherous night air would give you a chill"; so, despite the patient's pleadings, with dragon-like severity, she kept the door shut and the window almost hermetically sealed. As there is no doubt that such absolutely erroneous notions still exist, let me first state emphatically that fresh air never gives a chill. Exposure to cold may predispose to chill by lowering the resisting power of a part which then permits the intrusion of microbes, but the fresh air plays no part in causing the chill.

Consider the case of two men who both sleep out in the open, with no roof but the sky, and no bed but the ground. One is absolutely sober. He sleeps and wakes refreshed and wonders how anyone has ever been persuaded to take his slumber in a shut-up room. The other is drunk. He falls into the deep unrefreshing sleep of the intoxicated. Owing to the alcohol all his superficial vessels are dilated, while his internal organs, deprived of a normal supply of blood and previously vitiated by alcoholic poisoning, are in a fit state to invite all microbic invasion. Is it any wonder that an attack of acute nephritis, pleurisy, or pneumonia follows? The fresh air obviously has nothing to do with the matter, yet nevertheless it is all put down to the night air. Exposure to cold is one factor, alcoholic toxæmia is another, but the fresh air is blameless. If he had slept his drunken sleep on the stone cold floor of an airless kitchen, the after effects would doubtless have been the same, or perhaps even worse owing to the stuffiness.

Again, take the case of an old lady who leaves her close apartment and takes a walk abroad in fresh frosty air. Soon she is laid up with bronchitis, and she blames the much maligned pure fresh air, when she ought rather to blame the hot stuffy room, which dilated her superficial vessels with its temperature, as well as poisoned her system with its vitiated atmosphere. And thus, when she was exposed to cold, her lowered resistance could not withstand the invasion of bacteria.

Similarly, if anyone goes out shortly after taking a hot bath, or neglects to wrap up after becoming heated with hard exercise as from a game of football, the superficial vessels being dilated, the internal parts anæmic, exposure to cold may cause a "chill," whether it take the form of follicular tonsillitis, nasal catarrh, or otherwise. And this chill, like every other chill, is in no way due to the fresh air, though it may be erroneously put down to it.

After these few remarks, which I hope tend to clear the air from all false imputations previously laid at its door, it might be well to consider the composition and action of the atmosphere.

### The Composition of the Air.

The air consists approximately of one part of oxygen to four parts of nitrogen, with a small quantity of carbon dioxide. Nitrogen is an inert gas, and serves as far as the respiration of human beings is concerned, as a neutral medium to dilute in right proportion the active principal oxygen. The oxygen in respiration combines with the hæmoglobin of the blood to form oxy-hæmoglobin, which gives the brilliantly red tint to the arterial blood. The oxy-hæmoglobin gives up its oxygen to the tissues, and they, in turn, on combustion, return carbon dioxide to the blood which renders it blue and venous, and which escapes in the expired air. Inspired air contains 20.80 per cent. of oxygen and .04 of carbon dioxide. Expired air contains 16.00 per cent. of oxygen and 4.38 of carbon dioxide.

Thus air which is being breathed is losing its oxygen and becoming laden with carbon dioxide. Oxygen, besides being necessary for respiration, is a powerful disinfectant of the atmosphere, as it combines with injurious organic particles which may be suspended in the air, especially about towns and cities, and oxidising these particles renders them inert and harmless. Hence plenty of fresh air is indicated as a purifier in cases of zymotic disease. The fact that sanitas, Condy's fluid, and potassium chlorate owe their disinfectant property to their oxidising action shows clearly the power of oxygen and the need for it. That nascent oxygen bleaches serves to show its extreme activity, and in consequence peroxide of hydrogen has been used to remove pigment marks, but nascent oxygen does not exist in the atmosphere. Ozone is a very active form of oxygen found at sea, in high mountainous districts, and sometimes in the neighbourhood of electrical works. It is not only because of the ozone that patients are sometimes recommended to take a voyage, but also because at sea there are no microbes, no dust, and no particles of suspended organic matter, also the dull monotony of the boundless ocean may help to bring about the rest-cure by prohibiting excitement and inflicting quiet rest.

With regard to the stimulating effect of oxygen inhalations, little need be said. Most nurses have seen its wonderful effect in cases of collapse from pneumonia, and the soothing sleep it induces when administered to a "blue-baby" in convulsions.

### The Human Need of Air.

In a sick-room fresh air is of paramount importance both for respiration and as a disinfectant. The human need for fresh air is shown most clearly by the attempt to do without it. Absolute lack of air produces death by suffocation in a few minutes, while chronic insufficiency of air leads to a generally depressed state of health.

Oxygen is one of the essentials for metabolism. Take, for instance, the muscular system. If this is not supplied with well-oxygenated arterial blood, the chemical processes cannot go on satisfactorily, combustible products accumulate, clogging the tissue, the muscle fibre becomes lazy; it lacks tone and the common phenomenon known as lassitude results. Then perhaps tonics and massage or high frequency and Weir-Mitchell treatment are resorted to, and we forget that indispensable adjunct of health—"fresh air." The heart is often the first to suffer from "stuffiness," and a tendency to collapse, fainting, and nausea is a result not infrequently observed.

The nervous system, similarly to the muscular system, exhibits, when subjected to an atmosphere charged with carbonic acid and deficient in oxygen, distinct inertia, and undergoes a marked soporific effect. It is difficult to read, to think, or do any kind of mental work under such adverse circumstances, and, on the other hand, the languid drowsiness produced will not lead to a refreshing sleep, while a chronic state of such things may give rise to that most pernicious ill "insomnia."

Besides nervous depression and muscular depression, or perhaps consequent on these, the appetite becomes poor, and then dyspepsia, emaciation, and general irritability of temper are likely results. It is only a logical sequence, that if oxygen is lacking for combustion there is little use in piling on the fuel, and so no natural wish for food. Yet if the stuffy person be also greedy, he will probably manage to consume a good deal of fuel which is stored up as useless fat. Anorexia, dyspepsia, lack of muscular tone, and nervous prostration may be minor ailments, but a prolonged state of this leads to the storing up of toxins. A muddy complexion, a furred tongue, twinges of gout, intermittent headaches, and a general, thoroughly unhealthy condition little by little appears, making a wreck of the previously human creature. I have seen a pale, pasty, anæmic, over-worked student come up and ask the resident physician to sign the prescription of an iron tonic. "All right," he would say, "but fresh air is the tonic that you want." We all know the benefit of a month at the seaside, or in some bracing mountainous region where the air is freshest and purest, where the breezes are constantly changing the atmosphere, where smuts and microbes are "non sunt."

Fresh air stimulates respiration; it stimulates the appetite; it brings a healthy glow to cheek, and a general look of life and vigour. The rapid circulation of oxygen burns up waste products and toxins, and keeps the tissues clean, with the result that the complexion is clear, the eye is bright, and the intellect on the alert.

### The Ventilation of a Sick Room.

As patients, who have to lie in bed, cannot be nursed in the open in our English climate, it may be as well to point out how the air in the sick-room can be kept pure and fresh.

Firstly, the air in the room must be kept free from dust and suspended organic particles; and, secondly, a constant exchange of air from outside must be brought about. No carpets or heavy hangings should be allowed, as they harbour dust and microbic growth. Light washing fabric serves best for curtains, allowing of more perfect cleanliness. A polished floor rubbed over daily with a damp duster is advisable, and in this way the dust is kept from flying about. The furniture should be of the simplest, and no unnecessary pieces countenanced, as they likewise collect dust. For the wall, distemper colouring is better than a wall-paper, as it gives off no poisonous particles, does not distract the patient with its pattern, and it allows of washing. Another factor in keeping the air pure is a spray of weak carbolic or sanitas, which, when scattered about, disinfects the air.

With regard to the ingress of fresh air, two points are to be noted. The temperature of the room must be kept equable, not falling below 60° F. No draughts are permissible. As a rule, air enters by the window or door, and escapes by the chimney flue, and as these various apertures are usually to be found respectively situated on three different sides of a room, there need be little difficulty in ventilating a room, though there will probably be some in avoiding a draught. Still, a little thought and careful manipulation can, as a rule, prevent this. Almost all the year round in England a sick-room is the better for a fire, as it promotes the exchange of air as well as keeping the atmosphere warm. If possible a window in the room should be open, and the patient, if necessary, can be shielded from any slight draught by a screen. A window open at the top causes less perceptible draught than one open at the bottom, for the incoming fresh air mixes with the warm air in the upper strata of the room, and thus its temperature is raised before it is diffused. If the open window is too much, a Hincke's-Bird's window ventilator is to be recommended. This simple contrivance consists of a solid block of wood, which fits under the entire length of the lower window sash, and raises it so that a current of air directed upwards is admitted between the two sashes. When an open window in the room causes a draught, or lowers the temperature too much, an open window outside the room and the door left slightly ajar may serve for ventilation.

A great point in keeping the room wholly fresh is that the incoming air should be thoroughly diffused, and not pass directly from inlet to outlet. If, however, in the sick-room there is an alcove or recess where it is difficult, without an electric fan, to promote the circulation of air in that part, the nurse will be well advised not to have the patient's bed in that alcove, though doubtless she would think it out of draughts there. A Principal of a college, where some of the students had bed-sitting-rooms with the beds in curtained-off alcoves, told me how she noted new students in those rooms grow paler and paler, and how she was very glad when she was able to persuade her Council to do away with these alcoves.

I have not touched on the lighting of sick-rooms and the advantage of electricity over gas, nor of the heating of incoming air over steam-pipes, or hot water-pipes, for those are matters which the architect arranges. I have just taken an absolutely ordinary room of an English house, and tried to show how the nurse can keep it ventilated to the best advantage.

### Everybody's Opinion.

### THE APPOINTMENTS TO THE SOUTH-EASTERN FEVER HOSPITAL.

The matron of the Royal Victoria Hospital, Belfast, writes : " Miss E. Hothersall and Miss C. J. Pugh were not trained in the Royal Victoria Hospital, Belfast, as stated in your last issue, but they have worked here as staff nurses for the past year."

### AN APPEAL FROM NAZARETH.

" The Matron " of the British Hospital, Nazareth, Palestine, writes : Would any nurse whose days of study for examinations are a thing of the past and who has some old anatomy and nursing books put away and very seldom used, like to help some native nurses who are trying hard to master anatomy without the aid of books? They receive lectures from the doctor, but greatly need books to read up after and to turn to for reference on any point upon which they are not quite clear. They have all been educated at the Church Missionary Society's Orphanage, and are able to read and write English well. And they are also really desirous of becoming good nurses.

[Any readers desirous of helping had better communicate with the matron at Nazareth before sending books out.—Ed. Hospital.]

### ARMY NURSING.

" R. F." writes : I was sorry to see the statements of " J. M." in your valuable paper of July 28. I am an orderly in the Royal Army Medical Corps and I believe in justice everywhere. I cannot see where the different footing comes in, perhaps " J. M." can enlighten me. I admit that we have some sisters who are not very nice, but when there are very good sisters to work with, some of the orderlies neglect their work and then the sister gets the heavy end of the stick. Can you blame a sister if she is cross in such circumstances? I have been in an enteric ward and at first the sister did the work and I assisted, but since I was taught all the principal points in nursing this class of illness, I have done the work and the sister assisted. There are always two nurses to one patient. It is through teaching the orderlies ways of nursing and trusting them to carry them out that the sisters can do the " decorating," as " J. M." calls it. I now come to the point where the orderlies get all the praise. It is given to them indirectly, for I firmly believe that if it were given directly the orderlies would soon get a bit proud and could not be told if they went wrong. As a rule, the sisters have a more tender touch than the orderlies. Some of the latter, however, learn to handle gently, but it is not these orderlies who complain. As a rule, it is those men who have not been under a sister. Can you call that honest? I do not want to run down the orderlies of the Royal Army Medical Corps, for it would be unfair to do so; but the value of the sisters can be seen at any time in the acute wards. Does " J. M." think that he would be better off if he came under a ward master? I myself am afraid that, under a non-commissioned officer, the orderly's life would not be a very comfortable one. Is it not better to be under a sister who has had not less than three years' training? Indeed, if I mistake not, it is very rare to get one with only three years' training. Let every man speak as he finds things; it is more honourable.

### Appointments.

Borough Sanatorium, Bridlington.—Miss Clara Page has been appointed nurse matron. She was trained at the General Infirmary, Leeds, and the Borough Hospital, Wolverhampton. She has since been assistant nurse at the Birmingham Eye Hospital, staff nurse at the Memorial Hospital, Kendal; head nurse at the City Fever Hospital, Bradford; nurse matron at the Sanatorium, Morecambe; and superintendent nurse at the Wolstanton and Burslem Union Hospital.

City Hospital, Fazakerley, Liverpool.—Miss Katrine Ediss has been appointed assistant matron. She was trained at Little Bromwich Fever Hospital, Birmingham, Belvidere City Fever Hospital, Glasgow, and the Western Infirmary, Glasgow. She has since been night superintendent at Hull.

Croydon Union Infirmary.—Miss A. E. Tomkins and Miss F. Hopkins have been appointed ward sisters. Miss Tomkins was trained at Brentford Union Infirmary, and has since been sister at Kingston-upon-Thames Union Infirmary. Miss Hopkins was trained at St. Pancras Infirmary, and has since been charge nurse at the Western Fever Hospital, Fulham, and sister at Kensington Union Infirmary.

Dartmouth and Kingswear Cottage Hospital.—Miss Annie Harris has been appointed matron. She was trained at the City of London Hospital, Victoria Park, E., and the Great Northern Central Hospital, Holloway Road, N. She has since held appointments at the Hospital for Nervous Diseases, Maida Vale, W., the Victoria Hospital for Children, Chelsea, and Huddersfield Infirmary.

Gravelly Hill Workhouse Infirmary, Birmingham.—Miss Emma Jane Helliwell has been appointed charge nurse. She was trained at the Union Infirmary, Leeds.

Infirmary for Children, Liverpool.—Miss Ethel J. Atkins has been appointed lady superintendent. She was trained at St. Bartholomew's Hospital, London, and has since been matron of Cuddington Hospital, matron of the Park Hospital, Hither Green, matron of Shoreditch Infirmary, and matron of the Royal Hospital, Portsmouth.

Isolation Hospital, Melton Mowbray.—Miss H. Scott has been appointed staff nurse. She was trained at Brownlow Hill Hospital, Liverpool, and has since been attached to the Trained Nurses' Institution at Leicester.

King Edward VII.'s Sanatorium, Midhurst.—Miss E. I. E. Jones has been appointed assistant matron. She was trained at St. Thomas's Hospital, London, and has since been assistant matron at the Brompton Hospital Sanatorium at Frimley. She has lately been in charge of St. Thomas's Home for paying patients.

Leeds Hospital for Women and Children.—Miss Mary E. Turner has been appointed charge nurse. She was trained at Keighley Union Infirmary, and has since been charge nurse at Deanhouse Union Infirmary, Huddersfield, and staff nurse at the Leeds Hospital for Women and Children. She holds the certificate of the Central Midwives Board.

Poor Law Infirmary, Stapleton, Bristol.—Miss Caroline Jane Butcher has been appointed charge nurse. She was trained at Wolverhampton Union Infirmary, and has since been charge nurse at Hunslet Union Infirmary. She has also done private nursing.

Queen Victoria Memorial Hospital, Mont Boron, Nice.—Miss A. M. Hay has been appointed matron superintendent. She was trained at the Leith General Hospital and the Edinburgh Royal Maternity and Simpson Memorial Hospitals. She has since been sister at Leith General Hospital, night charge nurse at Chesterfield, sister at Dumfries and Galloway Royal Infirmaries, and matron of Cardiff Private Patients' Hospital.

South-Eastern Hospital, New Cross, E.C.—Miss E. Moore has been appointed charge nurse. She was trained at West Ham Infirmary, Leytonstone, London, and at Upney Fever Hospital, Barking, where she has since been charge nurse and deputy matron. She has also been sister at the City Hospital, Birmingham.

Training Ship "Mercury," Hamble, Southampton.—Miss Esther Grierson has been appointed sister-matron of the hospital of this institution. She was trained at the Longmore Hospital, Edinburgh, and the Great Northern Central Hospital, London. She has since been doing private work for the Blackheath Nursing Institution.

### Presentations.

City Hospital, Old Swan, Liverpool.—Miss Lilla L. S. Dawson having resigned her position of matron of the City Hospital, Old Swan, Liverpool, has been presented by the medical and nursing staff with a silver Queen Anne tea-service, and by the domestic staff with a silver cake-basket. She was also the recipient of several gifts from others connected with the hospital.

Lichfield Workhouse Infirmary.—Miss E. S. Butler, superintendent nurse, of Lichfield Workhouse Infirmary, was presented with a salad-bowl and biscuit-box by the officers of the Workhouse, on the occasion of her marriage, which takes place shortly. The matron in a few pleasant and suitable words made the presentation.

Nottingham and Notts Private Nursing Institution. Miss Gertrude Jenkins was presented with a silver toilet table-set by her friends at the Nottingham and Notts Private Nursing Institution, and with a pair of silver vases by the matron, on the occasion of her marriage.

### Novelties for Nurses.

(By our Shopping Correspondent.)

### LEMCO NEW DINNER AND LUNCHEON DISHES BOOKLET.

The value of Lemco as a preparation is well known, and a dainty little booklet issued by the Lemco Company, 4 Lloyd's Avenue, E.C., contains a number of novel recipes for using it. These will be found acceptable to cooks in search of absolutely original and exceedingly tempting dishes, whose name is legion. Many of the recipes have the advantage of being illustrated, and the whole of them are so admirably clear that he or she who runs may read.

### The Nurses' Bookshelf.

Nursing in the Acute Infectious Fevers. By Dr. George P. Paul. (Philadelphia and London: W. B. Saunders Co. 1906. Pp. 200. 4s.)

This admirably arranged and well printed book contains a large amount of useful information on the acute infectious diseases, including pneumonia and acute rheumatism. The first part treats of fever in general, with chapters on diet, reduction of temperature, and hygiene of the sick-room, etc.; and in the second part the various diseases are dealt with individually. An appendix is added with brief chapters on bacteriology, antiseptics, examination of urine, etc. Throughout the information is accurate, and the advice given is generally sound, but the author is not always careful to distinguish between the duties of nurse and doctor. For instance, he states that in scarlet fever the mouth and throat should be douched with some mild antiseptic, and mentions several; but he does not describe how this is to be done, although it is much more important for the nurse to have a knowledge of the practical details than to know the names of some antiseptics and the strength in which they should be used. Again, while describing the treatment of shock, he says: "Give stimulants by hypodermic, as strychnine, atropin, nitroglycerin, digitalin, and supra-renal solution." Any nurse reading this sentence might assume that she is entitled to do this without instruction from the doctor—a mistake which would probably get her into serious trouble. In fact, drugs and theoretical considerations occupy a somewhat too prominent place in a book intended for nurses, and there is not enough precision in defining what a nurse may do on her own initiative, and what she must report to the doctor and wait for his instructions. In the chapter on diphtheria the nursing of tracheotomy cases and during paralysis is unfortunately not described, and there is no mention made of paralysis of the diaphragm or of the precautions necessary to guard against heart-failure. However, in spite of some omissions the book is in many respects excellent, and will prove of much interest and service to nurses with some experience.

### TRAVEL NOTES AND QUERIES.

By our Travel Correspondent.

Pilgrimage to the Holy Land (A. H.).—I am sorry that I cannot help you at all in this matter; it is a purely private enterprise, and one of which I know nothing. You will receive full information if you write to the headquarters of the Assumptionist Fathers, and your own priest will be able to give you their address.

Brittany and Devonshire (J. S. R.).—So glad my recommendations have been so useful. Let me hear on your return how you liked St. Malo.

Cornwall or Devon (Spero).—I am afraid every place will be full the end of this month, but you will have a better chance in September, and it is a lovely month in those counties. At your terms, the best addresses I can give you are Miss Snell, Ivy Cottage, Caerhays, near St. Austell, Cornwall, and Miss Dolling Smith, The Homestead, Ringmore, near Kingsbridge, S. Devon.

**Rules in Regard to Correspondence for this Section.—All questioners must use a pseudonym for publication, but the communication must also bear the writer's own name and address as well, which will be regarded as confidential. All such communications to be addressed "Travel Correspondent, 28 Southampton Street, Strand." No charge will be made for inserting and answering questions in the inquiry column, and all will be answered in rotation as space permits. If an answer by letter is required, a stamped and addressed envelope must be enclosed, together with 2s. 6d., which fee will be devoted to the objects of "The Hospital" Convalescent Fund. Ten days must be allowed before an answer can be published.**

### Notes and Queries.

**REGULATIONS.**

**The Editor is always willing to answer in this column, without any fee, all reasonable questions, as soon as possible.**

**But the following rules must be carefully observed.**

1. **Every communication must be accompanied by the name and address of the writer.**
2. **The question must always bear upon nursing, directly or indirectly.**

**If an answer is required by letter a fee of half-a-crown must be enclosed with the note containing the inquiry.**

*Canada or America.*

(220) Should you advise a three years' certificated nurse to take up work in New York or Canada? Please give addresses of homes or hospitals.—*M. A. R.*

The supply of nurses in Canada appears to be adequate, and unless you have friends with influence we do not think you would easily secure employment in Canada or New York. The Montreal General and the Royal Victoria Hospital, Montreal; the Winnipeg General Hospital, Manitoba, Canada; the New York City Training School for Nurses, Blackwell's Island, New York; and Philadelphia Hospital, are a few addresses that may help you.

*Medico-Psychological Society.*

(221) Will you give me information as to the Medico-Psychological Society's certificate to male nurses? What is the youngest age a man can train as a male nurse?—*B. D. V.*

The address of the Society is 11 Chandos Street. Write to them for all information. There is no training school for civil male nurses except at the National Hospital for the Paralsed, Queen Square, Bloomsbury. Write there for full details.

*Advice Wanted.*

(222) Can you help me how to cure a boy of four years of age who wets his bed. The doctor says there is nothing wrong.—*Bloomer.*

We can only suggest that if the advice of one doctor fails, to ask another medical man.

*Massage.*

(223) Will you give me the names of institutions teaching massage and district nursing?—*Ascot.*

Write to the Society of Trained Masseuses, 12 Buckingham Street, Strand, for massage. You cannot secure training as a district nurse without joining the Queen Victoria Jubilee Institute for District Nurses, or becoming the pupil of a district nurse, which would be a private arrangement.

*District Maternity Work.*

(224) I have been inconvenienced by the non-attendance of a district nurse at maternity cases. Is this refusal to attend usual?—*Kappa.*

It is a mistake to permit a district nurse to attend maternity cases at all, and certainly she cannot be expected to do so when occupied with other serious general cases. Unfortunately some committees permit their nurses to attend maternity cases when free to do so, but this custom is wrong, and gives rise to many difficulties.

*Male Nurse.*

(225) Can you tell me where to apply for a situation as male nurse in South Africa? Private.—*S. Y. Z.*

We do not give the addresses of private institutions, and we know of no association in South Africa which employs male nurses.

*Ambulance.*

(226) We cannot answer *D. G. V.'s* anonymous query.

*Cottage Hospital.*

(227) Can you tell me the number of nurses required in a cottage hospital of 40 beds, averaging 26 to 30 patients daily?—*Alex.*

Will you tell us the number of wards, and whether there are any surgical cases?

**Handbooks for Nurses.**

| | Post Free. |
|---|---|
| "A Handbook for Nurses." (Dr. J. K. Watson) ... | 5s. 4d. |
| "Nurses' Pronouncing Dictionary of Medical Terms" | 2s. 0d. |
| "Art of Massage." (Creighton Hale.) ... ... ... | 6s. 0d. |
| "Surgical Bandaging and Dressings." (Johnson Smith.) ... ... ... ... ... ... | 2s. 0d. |
| "Hints on Tropical Fevers." (Sister Pollard.) ... | 1s. 8d. |

Of all booksellers or of The Scientific Press, Limited, 28 & 29 Southampton Street, Strand, London, W.C.

### For Reading to the Sick.

LED BY THE SPIRIT.

Conduct me safe, conduct me far
From every hurtful sin and snare;
Lead me to God, my final Rest,
In His enjoyment to be blest.

Lead me to holiness the road
That I must take to dwell with God;
Lead to Thyself, the Spring from whence
To fetch all quickening influence.

*Simon Browne.*

He is ever waiting till His servants are willing to be led on by Him to something better than they have yet attained to; not constraining them, but waiting to be constrained by them; leading out their unformed wishes into definite acts, and ever crowning those acts by further revelations of Himself. Thus He is constrained, but it is by the love of Himself which He Himself first kindles within.—*Isaac Williams.*

We must never allow the despairing thought, the darkness of doubt, to intervene to question the fulness of His grace. God may work in whom He wills, and we may surely believe that He will work when He comes to dwell, not as a passing tenant, but as an abiding possessor. He will make that heart which is to be His Home in all things pleasing to Himself. All is possible where He wills to be in order to give life. We must needs become what He wills in His time, however faulty and imperfect we may be in ourselves. —*T. T. Carter.*

Why fearest thou to take up the Cross which leadeth thee to a kingdom? In the Cross is salvation, in the Cross is life, in the Cross is protection against our enemies, in the Cross is all heavenly sweetness, in the Cross is strength of mind and joy of spirit, in the Cross the height of virtue, the perfection of sanctity.

There is no salvation of the soul, nor hope of everlasting life, but in the Cross.

Take up therefore thy cross and follow Jesus, and thou shalt go into life everlasting. He went before, bearing His Cross, and died for thee on the Cross, that thou mayest also bear thy cross, and desire to die on the Cross with Him.—*Thomas à Kempis.*

'Tis my happiness below
Not to live without the Cross,
But the Saviour's power to know,
Sanctifying every loss.
Trials must and will befall,
But with humble faith to see
Love inscribed upon them all,
This is happiness to me.

God in Israel sows the seeds
Of affliction, pain, and toil;
These spring up and choke the weeds
Which would else o'erspread the soil:
Trials make the promise sweet,
Trials give new life to prayer;
Trials bring me to His Feet,
Lay me low, and keep me there. *Cowper.*

